# Novel insights into innate sensing of HIV-infected cells

**DOI:** 10.1186/1742-4690-8-S2-O13

**Published:** 2011-10-03

**Authors:** Olivier Schwartz

**Affiliations:** 1 Institut Pasteur, Virus and Immunity Unit, URA CNRS 3015, 75724 Paris, France

## 

Our research is aimed at understanding the interplay between viruses and the immune system. We are analyzing how HIV infection triggers production of type-l interferon (IFN) and other cytokines in various cell types. Cell-free HIV-1 virions are considered to be poor stimulators of IFN production. We examined how HIV-infected cells are recognized by plasmacytoid dendritic cells (pDCs), by monocyte-derived DCs (MDDCs), and by other cells. We show that infected lymphocytes are more potent inducers of IFN than virions. There are target cell-type differences in the recognition of infected lymphocytes. In primary pDCs and pDC-like cells, recognition occurs in large part through TLR7, as demonstrated by the use of inhibitors and by TLR7 silencing. Donor cells expressing replication-defective viruses, carrying mutated reverse transcriptase, integrase or nucleocapsid proteins induced IFN production by target cells as potently as wild-type virus. In contrast, Env-deleted or fusion defective HIV-1 mutants were less efficient, suggesting that in addition to TLR7, cytoplasmic cellular sensors may also mediate viral sensing. Furthermore, in a model of TLR7-negative cells, we demonstrate that the IRF3 pathway, through a process requiring access of incoming viral material to the cytoplasm, allows sensing of HIV-infected lymphocytes. We are also currently analyzing the role of SAMHD1, a recently identified anti-HIV restriction factor, in the activation of MDDCs by HIV. Altogether, our results indicate that detection of HIV-infected lymphocytes occurs at different levels, through both endosomal and cytoplasmic pathways. Characterization of the mechanisms of innate recognition of HIV-infected cells allows a better understanding of the pathogenic and exacerbated immunologic events associated with HIV infection.

